# Prevalence of hyperthyroidism, hypothyroidism, and euthyroidism in thyroid eye disease: a systematic review of the literature

**DOI:** 10.1186/s13643-020-01459-7

**Published:** 2020-09-01

**Authors:** Juliana Muñoz-Ortiz, Maria Camila Sierra-Cote, Estefanía Zapata-Bravo, Laura Valenzuela-Vallejo, Maria Alejandra Marin-Noriega, Pilar Uribe-Reina, Juan Pablo Terreros-Dorado, Marcela Gómez-Suarez, Karla Arteaga-Rivera, Alejandra de-la-Torre

**Affiliations:** 1 Escuela Barraquer Research Group, Escuela Superior de Oftalmología del Instituto Barraquer de América, Avenida Calle 100 No. 18A – 51, Bogotá, Colombia; 2 Research Group in Neurosciences NeURos, Escuela de Medicina y Ciencias de la Salud, Universidad del Rosario, Carrera 24 # 63C 69, Bogotá, Colombia

**Keywords:** Dysthyroid ophthalmopathy, Hypothyroidism, Hyperthyroidism, Euthyroid, Prevalence

## Abstract

**Background:**

Thyroid eye disease is an autoimmune disorder of the orbital retrobulbar tissue commonly associated with dysthyroid status. The most frequent condition is hyperthyroidism, although it is also present in hypothyroid and euthyroid patients. The prevalence of thyroid conditions in patients with thyroid eye disease had been previously evaluated; however, there is no consensus on a global prevalence. The study aims to estimate the prevalence of hyperthyroidism, hypothyroidism, and euthyroidism in patients with TED, through a systematic review of literature.

**Methods:**

We conducted a systematic review of the literature following the PRISMA guidelines, in MEDLINE, COCHRANE, EMBASE, Science Direct, and LILACS databases. Inclusion criteria were primary studies of patients with a diagnosis of thyroid eye disease made by an ophthalmologist or with diagnosis criteria, with measurement of thyroid function (TSH, T3, and free T4), and diagnosis of the primary thyroid condition. A quality assessment was made through the Joanna Briggs Institute Quality tools. Finally, we extracted relevant details about the design, the results, and the prevalence of thyroid disorders in thyroid eye disease.

**Results:**

The initial search revealed 916 studies, of which finally thirteen met inclusion criteria. Six studies were performed in Europe (Germany, Wales, and Spain), five in Asia (Iran, South Korea, Japan, and Singapore), one in North America (USA), and one in Africa (Ghana). The global prevalence, in patients of thyroid eye disease, was 10.36% for hypothyroidism, 7.9% for euthyroidism, and 86.2% for hyperthyroidism.

**Conclusions:**

Professionals should be aware that thyroid eye disease can be present in patients with a normal thyroid function. The assessment for these patients is based on orbital images; serum TSH, T3, and free T4; antibody levels as thyrotropin receptor antibodies; and thyroperoxidase levels. Additionally, we want to encourage research in this field in other regions of the world such as Latin America.

**Systematic review registration:**

PROSPERO ID CRD42020107167

## Background

### Rationale

Thyroid disease (TD) is a quite common condition worldwide. According to the American Thyroid Association, only in the United States of America (USA) reported 20 million Americans with some form of TD, and at least 12% will develop a thyroid condition during their lifetime. According to hormonal levels, the patients with TD can be classified into three different groups: hypothyroidism, euthyroidism, and hyperthyroidism [1].

Euthyroidism is defined as normal thyroid hormone production and serum levels [2]. Hyperthyroidism is a clinical condition in which thyroid hormones are synthesized excessively. The reported prevalence is 0.8% in the USA and 1.3% in Europe. It is seen more frequently in women and adulthood. The clinical manifestations usually involve several systems, for example, weight loss can be evidenced despite no appetite disturbance, limb tremor, tachycardia, and tachypnea [3]. On the contrary, hypothyroidism is the condition in which thyroid hormones are deficient. It has a higher prevalence that varies between 0.3 and 3.7% in the USA and 0.2 and 5.3% in Europe. It occurs more frequently in women over 65 years of age and it is commonly seen in patients with autoimmune diseases, such as type 1 diabetes mellitus and celiac disease, among others. The clinical manifestations are usually weight gain, fatigue, and cold intolerance [4].

Thyroid eye disease (TED) is an autoimmune disorder of the orbital retrobulbar tissue associated with dysthyroidism, mainly hyperthyroidism in Graves’ disease (GD), even though it is present in hypothyroid and euthyroid patients [5].

The prevalence of dysthyroidism in patients with TED has been previously evaluated; however, there is no consensus on a global prevalence, and the physiopathological effect of thyroid hormones on the onset and progression of TED has not been fully understood.

The thyroid-stimulating hormone receptor (TSHr) and insulin-like growth factor 1 (IGF-1) receptor on orbital fibroblasts are likely to be the most important autoimmune targets in the disease. It has been hypothesized that clinical phenotypes such as euthyroid or hypothyroid TED, or the predominance of muscle or fat enlargement, may be caused by the molecular signature of different anti-thyrotropin-receptor antibodies [6]. Also, previous studies had concluded that dysthyroidism (hyperthyroidism or hypothyroidism) is associated with more severe presentations of TED, recommending the assessment of thyroid function during antithyroid treatment and the management of TED [7].

The study aims to estimate the prevalence of hyperthyroidism, hypothyroidism, and euthyroidism in patients with TED, through a systematic review of literature.

### Description of the condition

TED is the most common autoimmune disease of the orbit [8]. The disease appears two to six times more frequent in young women, but severe cases occur more frequently in men older than 50 years [8].

In TED´s physiopathology, the orbit becomes infiltrated by B and T cells, activating genes involved in inflammation and tissue remodeling [9]. Orbital fat and extraocular muscles expand from accumulating hyaluronidase-digestible material and adipogenesis [6]. These events are mediated by interleukins 1β, 6, 8, and 16; tumor necrosis factor α (TNF-α); RANTES (regulated on activation, normal T cell expressed and secreted); and CD40 ligand. The action of these cytokines turns bone marrow-derived fibrocytes into CD34+ fibroblasts that further differentiate into myofibroblasts or adipocytes [10].

The CD34+ fibroblasts express low levels of TSHr and other thyroid antigen receptors and overexpress IGF-1 receptors [11–13]. Thyroid-stimulating immunoglobulins activate the IGF-1 receptor complex, leading to the expression of inflammatory molecules and glycosaminoglycan synthesis; this signaling can also be activated in adipocytes [10].

Clinical manifestations of TED mainly include eyelid retraction (90%), exophthalmos (62%), restricted extraocular motility (43%), eye pain (30%), tearing (21%), diplopia (17%), photophobia (16%), blurred vision (8%), and optic nerve dysfunction (6%) [14]. Patients with euthyroid/hypothyroid TED developed significantly less severe ocular symptoms, less active, and more asymmetrical disease than hyperthyroid patients [15].

TED management starts with the control of environmental factors to decrease the risk of disease progression, such as smoking cessation. Local management of TED includes ocular lubrication, conjunctival autograft, or even orbital decompression in severe cases. The oral glucocorticoids are recommended to be administered as prophylaxis for mild active TED when treated with radioactive iodine in patients with GD. Intravenous glucocorticoids are the first line of treatment for moderate-to-severe and active TED. Options for disease maintenance are many and include orbital radiotherapy, selenium, cyclosporine, azathioprine, mycophenolate mofetil, tocilizumab, or rituximab [16]. Recently, a new pharmaceutic molecule was approved for TED, teprotumumab. This is a fully human monoclonal antibody that attenuates signaling initiated at the IGF-1 receptor complex blocking pathologic immune responses [17].

### Description of the thyroid function measurement and the diagnosis of the primary thyroid condition

Thyroid function measurement is made from the level of hormones produced by the thyroid gland and the hormone that stimulates its production. The normal thyroid function is called euthyroidism, described by the American Thyroid Association as a normal range between 0.4 and 4.0 mU/L for thyroid-stimulating hormone (TSH) [18]. On the other hand, hyperthyroidism presents elevated free thyroxine (T4) and triiodothyronine (T3) and decreased TSH serum levels (0.0–0.4 mU/L) [3]. Besides, hypothyroidism is diagnosed with elevated serum concentration of TSH (above 4.0 mU/L) and decreased free T4 levels [18].

### How the thyroid function measurement and the diagnosis of the primary thyroid condition might work

By measuring the TSH, T3, and free T4 levels in TED patients, the thyroid function can be objectively evaluated and classified. The prevalence report of these conditions will allow the calculation of weighted prevalence.

### Why it is important to do this review

The prevalence of TED in hyperthyroidism is very well described because of its high prevalence. However, the low prevalence calculation in hypothyroid and euthyroid patients with the ophthalmological condition means that in clinical practice, these patients are initially ruled out for the TED study. Diagnosis could be in a late stage with a delay in treatment initiation and complications development.

## Objective

The study aims to estimate the prevalence of hyperthyroidism, hypothyroidism, and euthyroidism in patients with TED, through a systematic review of literature.

## Methods

### Protocol and registration

The present review was performed according to the Preferred Reporting Items for Systematic Reviews and meta-analysis (PRISMA) guidelines [19]. The protocol registration can be found under the PROSPERO ID CRD42020107167.

### Study design

The study design is a systematic review of literature evaluating the prevalence of primary hyperthyroidism, hypothyroidism, and euthyroidism in patients with TED diagnosis.

### Eligibility criteria

We included all published articles if (a) the abstract was available; (b) it contained original data; (c) the TED was diagnosed by an ophthalmologist or diagnosis criteria were settled; (d) the thyroid disorders were measured with blood levels of TSH, free T4, and T3 [3, 18]; and (e) if it reported the prevalence of primary thyroid function variation on TED patients. If the prevalence was not reported but was possible to calculate it, the study was also included. Articles were excluded from the analysis if the diagnosis of the thyroid function status were secondary to thyroid disease treatment or if the study design was a review, letter to the editor, case report, case series, or systematic reviews.

### Information sources

We used a combination of exploded controlled vocabulary (MeSH, Emtree, DeCS) and free-text terms (considering spelling variants, plurals, synonyms, acronyms, and abbreviations) with field labels, truncation, proximity operators, and Boolean operators.

The literature search was conducted in the following electronic databases up to July 29 of 2019: MEDLINE, COCHRANE, EMBASE, ScienceDirect, and LILACS. No limits regarding language and period of publication were used (Additional file 1). The search was updated on April 21 of 2020.

### Study selection

**Fig. 1:**
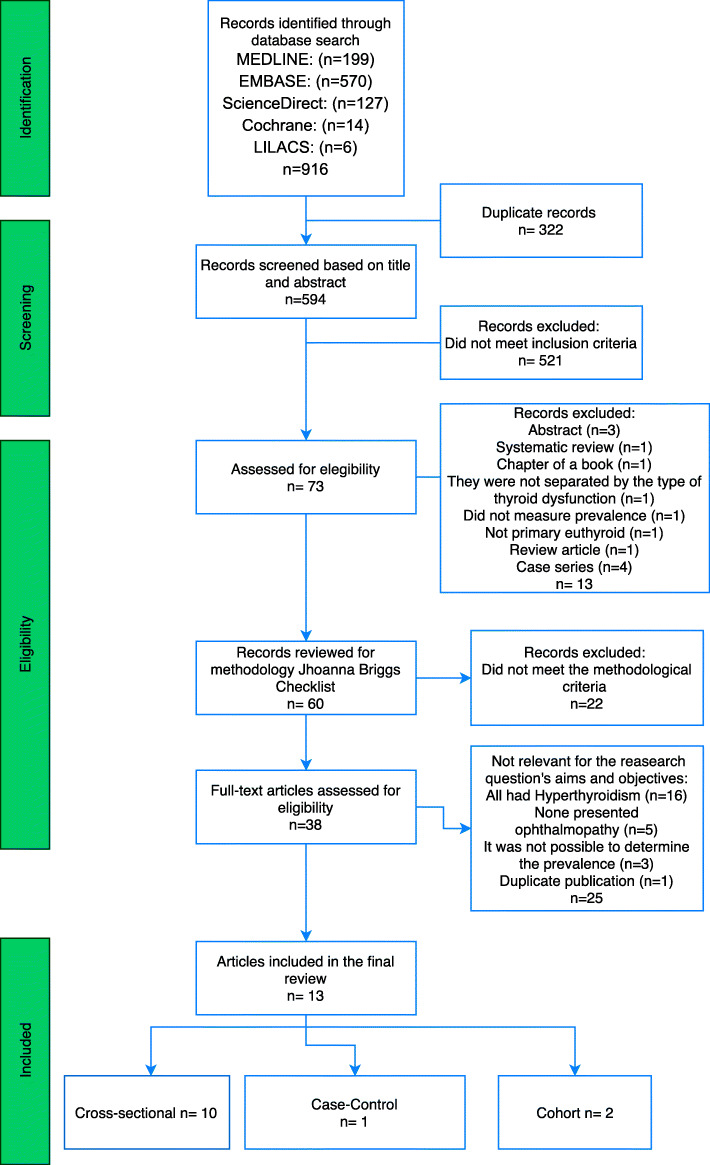
PRISMA flowchart

The electronic search was made by two reviewers (JMO and MCSC). Duplicates were eliminated through an Excel function. We performed an independent review of article titles and abstracts extracting data according to the predefined eligibility criteria. In case of disagreement, a third reviewer (ADLT) made the inclusion decision (Fig. 1).

### Data collection process

A data collection form was designed in Excel. Three reviewers (MCSC, EZB, and LVV) independently extracted relevant details about the design and the results of each study which included author, study period, location, study design, number of patients with TED, and the prevalence of primary hyperthyroidism, hypothyroidism, and euthyroidism.

### Risk of bias

We followed the chapter of systematic reviews of prevalence and incidence of the Johanna Briggs Institute (JBI) to conduct this systematic review and classified the articles using the respective tool for each study design [20, 21]. An expert methodologist (MGS) established cutting points for the minimum score acceptable for study inclusion: 72% for cohort studies, 75% for cross-sectionals, and 80% for case-control, or if any of it met all our major inclusion criteria [21] (Additional file 2).

## Results

### General description

Our search strategy extracted 916 published articles (199 from MEDLINE, 570 from Embase, 127 from ScienceDirect, 14 from Cochrane and 6 from LILACS).

After screening, 73 articles were assessed for eligibility and only 60 met the selection criteria and were evaluated through the JBI quality tool; 38 full-text articles were assessed for eligibility. Finally, after discarding 25 articles for different reasons (all the patients had hyperthyroidism, did not present ophthalmopathy, or it was not possible to determine the prevalence) 14 articles met all the inclusion criteria, but two articles had the same sample because they were made in the same population. To avoid overrepresentation of this cross-sectional study, we included only the data of one of them [22, 23] (Fig. 1) (Additional file 3).

### Characteristics

Our final inclusion strategy yielded 13 published articles, 6 were conducted in Europe (Germany, Wales, and Spain), 5 in Asia (Iran, Korea, Japan, and Singapore), one in North America (USA), and one in Africa (Ghana). All the studies were published between 1996 and 2018. Two of the studies had a small sample size of TED patients (18 and 47) and the rest had a bigger population (between 103 and 1020). All patients had a diagnosis of TED and thyroid function had been measured with TSH, free T4 levels, and T3 (if needed) or had it initially as an inclusion criteria (Tables 1 and 2).

**Table 1:** Selection criteria

Author	Study design	Endocrinological diagnosis of thyroid status	Ophthalmological diagnosis of TED	Exclusion of secondary to thyroid disease treatment thyroid hormone status at TED onset	Observations
Ackuaku-Dogbe et al. [24]	Cross-sectional	Thyroid function tests and physical examination	By ophthalmologist	Authors measured the thyroid status at the time the first treatment of the disease was established	NA
Expósito et al. [25]	Cross-sectional	Thyroid function tests	According to information register in clinical records	Information of basal hormonal status was extracted from the data of the cause of the disease (GD or Hashimoto thyroiditis)	This study only includes glucocorticoid-treated patients for an active moderate-severe TED status and does not explain through which criteria the patients were diagnosed with TED
Bartley et al. [14]	Cross-sectional	Thyroid function tests	By ophthalmologist and imaging tests	Authors measured the thyroid status diagnosis index registered on the medical records	The patients classified as primary hypothyroidism and Hashimoto thyroiditis (all patients were in hypothyroid status) were merged into the hypothyroid group to calculate the prevalence
Eckstein et al. [15]	Cohort study	Thyroid function tests	According to information register in clinical records	Investigators measured the thyroid status before or within 6 months after the onset of TED	NA
Jang et al. [26]	Case-Control	Thyroid function tests	By ophthalmologist and CT scans	The diagnosis of euthyroidism was based on normal serum hormone levels and no clinical history of hyperthyroidism. Hyperthyroidism was established using hormonal criteria and if the patient had a history of antithyroid therapy, in this last group only patients that remained hyperthyroid were included.	Hypothyroid patients were not included in the study
Kashkouli et al. [23]	Cross-sectional	Thyroid function tests, thyroid gland-scan, and sonography	By a specialized ophthalmological clinic	The classification of thyroid hormone status was made according to the primary thyroid diagnosis register on the clinical records	A limitation of this study is the underestimation of euthyroid patients. This study included patients from the thyroid disorder and not from the ophthalmological diagnosis, euthyroid patients were excluded from the beginning and therefore were not evaluated by ophthalmology
Khoo et al. [27]	Cohort study	Thyroid function tests	By experienced endocrinologists and for equivocal cases further evaluations were carried out by ophthalmology colleagues	The study did not mention possible causes of secondary hormonal status on the patients (following thyroid disease treatment)	The total number of patients with TED identified at the Thyroid Clinic was calculated according to the patients that were initially included in the study (1020). 1001 were excluded because they had previous history of thyrotoxicosis or hyperthyroidism (we included these patients into the hyperthyroid group), 10 had subclinical thyrotoxicosis (these patients were added to the hyperthyroid group), and the other 9 patients were in hypothyroid or euthyroid status
McKeag et al. [28]	Cross-sectional	Thyroid function tests	By ophthalmologist and imaging tests	The criteria of the study included only patients with Graves’ thyrotoxicosis before or at the time of diagnosis of TED and only patients with primary hypothyroidism are named in the study.	
Medghalchi et al. [29]	Cross-sectional	Thyroid function tests	By ophthalmologist, imaging tests and visual field analysis	The study did not mention possible causes of secondary hormonal status on the patients (following thyroid disease treatment)	
Mukasa et al. [30]	Cross-sectional	Thyroid function tests	By a specialized ophthalmological clinic	All the patients included were untreated and were assessed for GO within 3 months of their initial visit to the hospital	It is not clear if the prevalence of hypothyroidism was cero in this group or if at the beginning of the study these patients were excluded
Ponto et al. [31]	Cross-sectional	By a specialized autoimmune (endocrine) clinic	By independent ophthalmologist or a specialized thyroid–eye clinic	The primary hormone levels were measured according to the autoimmune-associated thyroid disease	A limitation of this study is the underestimation of euthyroid patients. This study initially included patients with autoimmune thyroid disease (GD and Hashimoto’s thyroiditis), euthyroid patients were not included in the initial selection criteria and were gathered with hypothyroid patients
Ponto et al. [32]	Cross-sectional	Thyroid function tests	By an ophthalmologist	The primary thyroid status was measured 6 months before or after TED diagnosis	NA
Cozma et al. [33]	Cross-sectional	By a specialized thyroid–eye clinic, thyroid function tests	By a specialized thyroid-eye clinic	Patients with normal thyroid function tests, no history of disease or treatment, no clinical signs or symptoms of thyroid disease were included	NA

**Table 2:** Characteristics of included studies

Author	Study period	Country	Number of patients with TED	Prevalence (%) hyperthyroidism in TED	Prevalence (%) hypothyroidism in TED	Prevalence (%) euthyroidism in TED
Ackuaku-Dogbe et al. [24]	2014–2016	Ghana	117	104 (88.9)	5 (4.3)	8 (6.8)
Expósito et al. [25]	2007–2011	Spain	18	12 (66.7)	6 (33.3)	–
Bartley et al. [14]	1976–1990	USA	120	108 (90)	5 (4.2)	7 (5.8)
Eckstein et al. [15]	2000	Germany	182	143 (78.6)	11 (6)	28 (15.4)
Jang et al. [26]	2008–2010	South Korea	163	139 (85.3)	–	24 (14.7)
Kashkouli et al. [22]	2003–2006	Iran	303	280 (92.4)	23 (7.5)	–
Khoo et al. [27]	1996–1999	Singapore	1020	1011 (99.1)	2 (0.2)	7 (0.7)
McKeag et al. [28]	2006–2007	Wales	47	44 (93.6)	3 (6.4)	–
Medghalchi et al. [29]	2012–2014	Iran	103	83 (80.5)	19 (18.4)	1 (0.9)
Mukasa et al. [30]	2010–2010	Japan	238	210 (88.2)	–	28 (11.8)
Ponto et al. [31]	1999–2012	Germany	610	584 (95.7)	–	–
Ponto et al. [32]	2005–2012	Germany	461	441 (95.6)	12 (2.6)	8 (1.7)
Cozma et al. [33]	1997–2005	Wales	140	92 (65.7)	29 (20.7)	19 (13.6)

### General description of the included studies

#### Cohort studies

Of the 13 studies, two were cohort studies [15, 27]. The first one, performed by Eckstein et al., reported on a retrospective study from a TED database 182 consecutive patients treated at the University Hospital of Essen, Germany [15]. The second one was made between 1996 and 1999 in Asia, the sample was of 1020 patients with TED diagnosis, and 1001 were excluded in their analysis study because they had the previous history of thyrotoxicosis or hyperthyroidism (we included these patients into the hyperthyroid group of our study). Ten had subclinical thyrotoxicosis (these patients were added to the hyperthyroid group in our study), and the other nine patients were in hypothyroid or euthyroid status [27].

#### Cross-sectional studies

Ten articles were cross-sectional studies and published between 1996 and 2018. One of the studies was conducted in the USA, and the rest in Wales, Germany, Spain, Iran, Japan, and Ghana. The population’s range within the studies was between 18 and 610 patients; the ten articles had individuals with TED and primary hyperthyroidism, with a calculated prevalence of 85.7% in the ranges of 65.7–95.7%. In eight articles, primary hypothyroidism was present in TED individuals, with a calculated prevalence of 12.18% between 2.6 and 33.3% and euthyroid patients with TED was present in six articles without a history of dysthyroidism, with a calculated prevalence of 6.7% with a range between 0.9 and 13.6%. Data from a referral endocrinology clinic (Tehran University Institute of Endocrinology) were overrepresented and two studies pertained to the same population.

#### Case-control studies

The case-control study published by Jang et al. determined the clinical and biochemical characteristics of 163 Korean patients with TED between 2008 and 2010 [26].

### Thyroid states

#### Hyperthyroidism

**Fig. 2:**
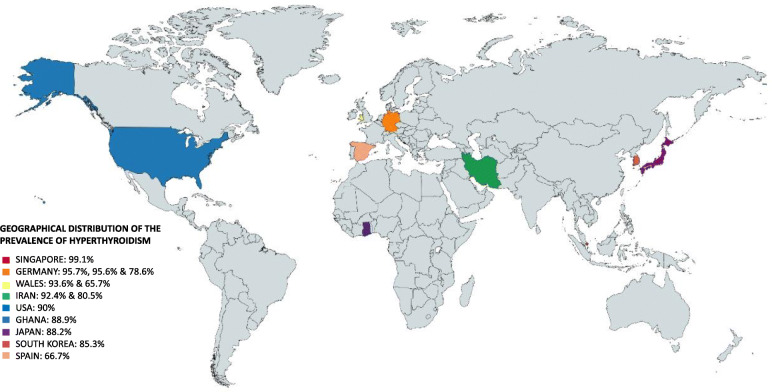
Geographical distribution of prevalence of hyperthyroidism (Singapore, Germany, Wales, Iran, USA, Ghana, Japan, South Korea, and Spain)

The thirteen studies reported the presence of primary hyperthyroidism in patients with TED. Some of the studies reported GD [25, 28, 30, 31] as the primary cause of hyperthyroidism. The range of prevalence was between (65.7–99.1%), with a total calculated prevalence of 86.2%. Countries like Wales (93,6%), USA (90%), and Iran (92,4%) had a higher prevalence, in contrast to another study in Wales with 65.7% and Spain that had the lowest prevalence (66.7%) (Fig. 2).

#### Hypothyroidism

**Fig. 3:**
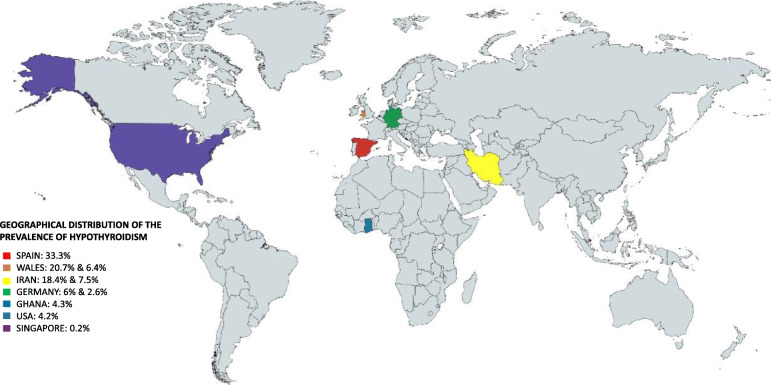
Geographical distribution of prevalence of hypothyroidism (Spain, Wales, Iran, Germany, Ghana, USA, and Singapore)

Ten studies (eight cross-sectional and two cohorts) reported the presence of primary hypothyroidism patients presenting TED, with a range of prevalence between 0.2 and 33.3% and a total calculated prevalence of 10.36%, where Spain showed the higher prevalence and Singapore the lowest. Some of them with the diagnosis of Hashimoto hypothyroidism [25]. All patients with secondary hypothyroidism (after treatment for any dysthyroidism with thyroidectomy, ion ablation, or radiation) were excluded (Fig. 3).

#### Euthyroidism

**Fig. 4:**
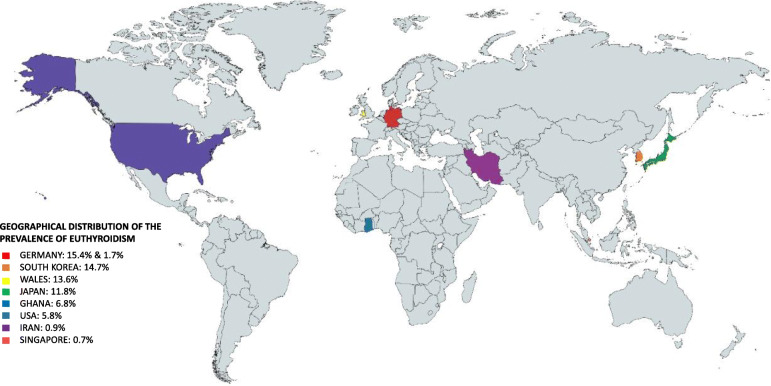
Geographical distribution of euthyroid prevalence (Germany, South Korea, Wales, Japan, Ghana, USA, Iran, and Singapore)

Nine of the 13 studies had TED patients with euthyroidism. The prevalence ranges from 0.9% in Iran and 15.4% in Germany. With a total calculated prevalence of 7.9%, the studies were located in Ghana, USA, Germany, Korea, Iran, Singapore, Japan, and Wales. The TED sample size ranges from 103 to 1020 patients (Fig. 4).

## Discussion

### Context

TD is a widely common endocrine pathology. According to the American Thyroid Association, nearly 12% of the USA population develop any thyroid condition during their life, and closely 20 million Americans have some form of TD. Thyroid disbalances can cause certain serious consequences, such as cardiovascular diseases, osteoporosis, complications during pregnancy, and infertility [1]. One important manifestation of TD is TED. This condition can significantly decrease the quality of life of those who suffer it due to its signs and symptoms [34].

The importance of this study lies in providing information about the level of thyroid hormones in patients with TED, to correctly differentiate Graves’ ophthalmopathy (GO) and TED. This confusion occurs since most patients with TED have biochemical evidence of hyperthyroidism with the most common cause being GD; however, TED may occur in patients with hypothyroidism (mainly Hashimoto’s thyroiditis) or euthyroidism [35].

### Methodology assessment

Many studies had been carried out studying TED. In our literature search, we found much information regarding this topic, including etiology, pathophysiology, diagnosis and evaluation, and treatment. A common premise, found in most of the studies, was the importance of an early diagnosis for a timely therapeutic approach and thus minimize the negative consequences for the patient. Besides, this disease appears to affect more women of reproductive age [8] and children and adolescents in a similar proportion or even slightly higher than adults [36].

Even though we found a lot of information to answer our question, we had to exclude most of the papers and studies we found, due to their lack of methodological quality. As explained in the methodology section, we used the Joanna Briggs guidelines to assess the methodological quality of each article we read. Only 63.33% of the studies were included after quality evaluation. Besides providing information about thyroid states in TED, we make a special call to the scientific community to take into account adequate scientific methodology using the standardized tools for each study design.

In our literature search, we identified different studies that allowed the measurement of the prevalence of thyroid disorders in TED. We hypothesized that possible causes were the range of the sample size on each study, their geographical location, or their epigenetic factors influencing the results. Similarly, we found that 16 studies included TED patients only associated with GD, underestimating the prevalence of hypothyroidism and euthyroidism.

### Clinical aspects

Hyperthyroidism is a thyroid disorder that has been known as the most prevalent associated with TED. In our study, the estimated prevalence was 86.2%. The most frequent cause of hyperthyroidism is GD. Patients present with heat intolerance, sweating, weight loss, goiter, emotional lability, insomnia, hyperkinetic behavior, fatigue, weakness, tachycardia, and tremors [37].

Even though TED and GD share multiple similarities, they are two separate conditions. Although both involve the immune system, especially TRAbs, the target organ is different, in TED is the eye and orbit, while in GD is the thyroid gland.

In GD, autoreactive T cells against the TSHr escape both central and peripheral selection [38]. B cells develop into antibody-producing plasma cells in a process requiring second signals interacting with T cells, thus resulting in the production of cytokines such as interleukins 1β, 6, and 12; interferon-γ; tumor necrosis factor α; CD40 ligand; and others. It promotes antibody secretion and T cell support of class switching [39]. After T cells have infiltrated the thyroid gland, thyroid epithelial cells express MHC class II molecules because of interferon-γ action. Thus, they have the potential to present thyroid antigens to T cells, perpetuating the inflammatory process [10]. Activated autoantibodies of the IgG1 subclass, primarily generated by intrathyroidal B cells, are directed against the thyrotropin receptor. These antibodies stimulate thyroid hormone production that is uncontrolled by the hypothalamic-pituitary axis [40].

Genetic determinants conferring susceptibility to GD have been identified. These include genes encoding thyroglobulin, thyrotropin receptor, HLA-DRβ-Arg74, the protein tyrosine phosphatase nonreceptor type 22 (PTPN22), and proteins involved in T cell signaling [41–43]. Epigenetic factors such as dietary iodine, exposure to tobacco smoke, infections, emotional stress, and alemtuzumab therapy are also associated with GD [44].

In an investigation made by Yin et al., genetic association studies were performed in a highly characterized GO population and compared with patients with GD but no clinically apparent GO. They found that the allele and genotype frequencies were not statistically different between GO and non-GO patients, concluding that GO does not have a distinct genetic susceptibility to their eye disease [45]. Further studies should evaluate the influence of immunologic, genetic, and epigenetic factors in GO and TED in hypothyroid and euthyroid patients.

Hashimoto thyroiditis (HT) is part of a spectrum of thyroid autoimmune diseases, ranging from typically self-limiting focal forms [46] to other several clinicopathologic entities like hashitoxicosis. Most HT forms ultimately evolve into hypothyroidism (with systemic clinical manifestations of a slow metabolism), although, in the initial presentation, patients can be hyperthyroid or even euthyroid [47]. Moreover, HT is associated with disturbance in genes like the TSHr and with IL1RNVNTR polymorphisms, and they also help as a prognostic indicator [48].

All forms of HT are characterized pathologically by lymphocyte B and T cell infiltration of the thyroid gland, as well as follicular helper T cells, increased in the thyroid peripheral blood. Furthermore, there is a clear correlation with antigen-specific T suppressor failure such as decreased sensitivity of CD4+ T cells to the inhibitory effect of TGFβ [46]. Antibodies found in HT not only make part of the pathogenesis of the disease but also help to establish a diagnosis and predict the development of hypothyroidism [46, 47].

Whereas thyroid-stimulating antibodies (TSAb) have a clear functional role activating TSHr in GD, it is also known the existence of blocking activity by the thyroid-blocking antibodies (TBAb). The balance between TSAb/TBAb determines disease presentation and fluctuating thyroid hormone levels between hyper or hypothyroidism in patients with thyroid autoimmune diseases [46].

Furthermore, few studies had demonstrated a strong association between functional TSAb and TED in patients with autoimmune HT. TSAb was highly prevalent in those with clinically overt and associated TED compared to HT patients without eye pathologies. Also, TSAb may be relevant to the pathophysiology of orbital involvement in HT [49].

The onset of the TED manifestation can be different between the hyperthyroid, hypothyroid, and euthyroid patients. Studies have demonstrated a lower involvement of the orbital-soft-tissue in patients with hypothyroid and euthyroid state [15]. Moreover, Jang et al. concluded that euthyroid patients have higher asymmetrical involvement (79.2%) than hyperthyroid patients (27.3%) [26].

### Limitations

The diagnosis method of TED was variable between the studies, and the information they provided about the selection criteria was based on an assessment made by an ophthalmologist or disease specialized center, as shown in Table 1. Hence, we recognize that a limitation of our study is the lack of standardization in TED diagnosis among the included studies.

A small number of included works geographically dispersed and highly variable “*n*” can be limitations of our work. Nevertheless, it was the result after the quality and data assessment of all the found articles.

## Conclusion

We recommend to ophthalmologists to be aware of TED clinical signs and suspect it even if the patients have a normal thyroid function. The assessment for these patients should be based on orbital images, serum T3, free T4, TSH, TRAbs, and interdisciplinary management with the endocrinologist.

There are many studies made in Asia, Germany, USA, and Wales, among other countries, but there is a lack of information about this disease in Latin American population. This is why we encourage researchers to obtain data from Latin American countries. Also, we strongly recommend performing primary studies following high-quality methodology tools for future investigations.

## Supplementary information

**Additional file 1.** Search.

**Additional file 2.** JBI Critical Appraisal Checklist for Cohort Studies.

**Additional file 3.** JBI quality tools results for final selected articles.

## Data Availability

The datasets used and/or analyzed during the current study are available from the corresponding author on reasonable request.

## Abbreviations

TD: Thyroid disease
USA: United States of America
TED: Thyroid eye disease
GD: Graves’ disease
TSHr: Thyroid-stimulating hormone
IGF-1: Insulin-like growth factor 1
TSH: Thyroid-stimulating hormone
T4: Thyroxine
PRISMA: Preferred Reporting Items for Systematic Reviews and meta-analysis
JMO: Juliana Muñoz Ortiz
MCSC: María Camila Sierra-Cote
ADLT: Alejandra de-la-Torre
EZB: Estefanía Zapata-Bravo
LVV: Valenzuela-Vallejo
MGS: Marcela Gomez-Suarez
TRAbs: Thyrotropin receptor antibodies
HT: Hashimoto thyroiditis
TSAb: Thyroid-stimulating antibodies
TBAb: Thyroid-blocking antibodies
