# Supplementary material for: Prevalence of hyperthyroidism, hypothyroidism, and euthyroidism in thyroid eye disease: a systematic review of the literature
Source: Syst Rev. 2020 Sep 1;9:201. doi: 10.1186/s13643-020-01459-7 (PMC7465839; doi:10.1186/s13643-020-01459-7)
Supplement: Supplementary file 1 — Additional file 1. Search. [file 13643_2020_1459_MOESM1_ESM.docx]

**Annex 1. Search**

**MEDLINE**

((((((((((Euthyroid[Title/Abstract]) OR Non Thyroidal Illness[Title/Abstract]) OR Normal functioning of the thyroid[Title/Abstract]) OR Normal thyroid hormones[Title/Abstract]) OR euthyroidism[Title/Abstract])) OR (((Hyperthyroidism[MeSH Terms]) OR Primary Hyperthyroidism[Title/Abstract]) OR Hyperthyroids[Title/Abstract])) OR (((((((Hypothyroidism[MeSH Terms]) OR Hypothyroidism[Title/Abstract]) OR Primary Hypothyroidism[Title/Abstract]) OR Thyroid-Stimulating Hormone Deficiency[Title/Abstract]) OR TSH Deficiency[Title/Abstract]) OR Secondary Hypothyroidism[Title/Abstract]) OR Central Hypothyroidism[Title/Abstract]))) AND (((((((((graves ophthalmopathy[MeSH Terms]) OR Graves Ophthalmopathy[Title/Abstract]) OR Dysthyroid Ophthalmopathy[Title/Abstract]) OR Thyroid Associated Ophthalmopathies[Title/Abstract]) OR Graves Orbitopathy[Title/Abstract]) OR Myopathic Ophthalmopathy[Title/Abstract]) OR Edematous Ophthalmopathy[Title/Abstract]) OR Congestive Ophthalmopathy[Title/Abstract]) OR Infiltrative Ophthalmopathies[Title/Abstract])) AND ((((((epidemiology[MeSH Terms]) OR epidemiology[Title/Abstract]) OR Prevalence[Title/Abstract]) OR Prevalence[Title/Abstract]) OR frequency[Title/Abstract]) OR global prevalence[Title/Abstract])

**EMBASE**

('euthyroidism'/exp OR 'hyperthyroidism'/exp OR 'hypothyroidism'/exp) AND ('endocrine ophthalmopathy'/exp OR 'graves ophthalmopathy':ti,ab,kw OR 'orbit disease'/exp) AND ('epidemiology'/exp OR 'prevalence'/exp) AND [embase]/lim AND [humans]/lim

**ScienceDirect**

(Euthyroid OR euthyroidism OR Hyperthyroidism OR Hyperthyroids OR Hypothyroidism OR Thyroid-Stimulating Hormone Deficiency OR TSH Deficiency) AND (graves ophthalmopathy OR Dysthyroid Ophthalmopathy OR Thyroid Associated Ophthalmopathies OR Graves Orbitopathy) AND (epidemiology OR Prevalence OR frequency OR global prevalence)

**Cochrane**

(Euthyroid OR Non Thyroidal Illness OR Normal functioning of the thyroid OR Normal thyroid hormones OR euthyroidism OR Hyperthyroidism OR Primary Hyperthyroidism OR Hyperthyroids OR Hypothyroidism OR Primary Hypothyroidism OR Thyroid-Stimulating Hormone Deficiency OR TSH Deficiency OR Secondary Hypothyroidism OR Central Hypothyroidism) AND (graves ophthalmopathy OR Dysthyroid Ophthalmopathy OR Thyroid Associated Ophthalmopathies OR Graves Orbitopathy OR Myopathic Ophthalmopathy OR Edematous Ophthalmopathy OR Congestive Ophthalmopathy OR Infiltrative Ophthalmopathies) AND (epidemiology OR Prevalence OR frequency OR global prevalence)

**LILACS**

(Euthyroid OR euthyroidism OR Hyperthyroidism OR Hyperthyroids OR Hypothyroidism OR Thyroid-Stimulating Hormone Deficiency OR TSH Deficiency) AND (graves ophthalmopathy OR Dysthyroid Ophthalmopathy OR Thyroid Associated Ophthalmopathies OR Graves Orbitopathy) AND (epidemiology OR Prevalence OR frequency OR global prevalence)
