# Supplementary material for: Prevalence of hyperthyroidism, hypothyroidism, and euthyroidism in thyroid eye disease: a systematic review of the literature
Source: Syst Rev. 2020 Sep 1;9:201. doi: 10.1186/s13643-020-01459-7 (PMC7465839; doi:10.1186/s13643-020-01459-7)
Supplement: Supplementary file 2 — Additional file 2. JBI Critical Appraisal Checklist for Cohort Studies. [file 13643_2020_1459_MOESM2_ESM.pdf]

THE JOANNA BRIGGS INSTITUTE

JBI Critical Appraisal Checklist for Cohort Studies

Reviewer____________________________________Date___________________________

Author _____________________________________Year_________Record Number______

	Yes	No	Unclear	Not applicable
1. Were the two groups similar and recruited from the same population?	✓	☐	☐	☐
2. Were the exposures measured similarly to assign people to both exposed and unexposed groups?	✓	☐	☐	☐
3. Was the exposure measured in a valid and reliable way?	✓	☐	☐	☐
4. Were confounding factors identified?	✓	☐	☐	☐
5. Were strategies to deal with confounding factors stated?	✓	☐	☐	☐
6. Were the groups/participants free of the outcome at the start of the study (or at the moment of exposure)?	✓	☐	☐	☐
7. Were the outcomes measured in a valid and reliable way?	✓	☐	☐	☐
8. Was the follow up time reported and sufficient to be long enough for outcomes to occur?	✓	☐	☐	☐
9. Was follow up complete, and if not, were the reasons to loss to follow up described and explored?	✓	☐	☐	☐
10. Were strategies to address incomplete follow up utilized?	✓	☐	☐	☐
11. Was appropriate statistical analysis used?	✓	☐	☐	☐

Overall appraisal: Include ☐ Exclude ☐ Seek further info ☐

Comments (Including reason for exclusion)

Major criteria: ✓

Minor criteria: ✓

Inclusion criteria: 8/11 (At least 7 meet the major inclusion criteria ✓)

© Joanna Briggs Institute 2017

THE JOANNA BRIGGS INSTITUTE

JBI Critical Appraisal Checklist for Case Control Studies

Reviewer____________________________________Date__________________________________

Author ______________________________________Year_________Record Number____________

	Yes	No	Unclear	Not applicable
1. Were the groups comparable other than the presence of disease in cases or the absence of disease in controls?	☑	☐	☐	☐
2. Were cases and controls matched appropriately?	☑	☐	☐	☐
3. Were the same criteria used for identification of cases and controls?	☑	☐	☐	☐
4. Was exposure measured in a standard, valid and reliable way?	☑	☐	☐	☐
5. Was exposure measured in the same way for cases and controls?	☑	☐	☐	☐
6. Were confounding factors identified?	☑	☐	☐	☐
7. Were strategies to deal with confounding factors stated?	☑	☐	☐	☐
8. Were outcomes assessed in a standard, valid and reliable way for cases and controls?	☑	☐	☐	☐
9. Was the exposure period of interest long enough to be meaningful?	☑	☐	☐	☐
10. Was appropriate statistical analysis used?	☑	☐	☐	☐

Overall appraisal: Include ☐ Exclude ☐ Seek further info ☐

Comments (Including reason for exclusion)

Major criteria: ✓

Minor criteria: ✓

Inclusion criteria: 8/10 (At least 5 meet the major inclusion criteria ✓)

THE JOANNA BRIGGS INSTITUTE

JBI Critical Appraisal Checklist for Analytical Cross Sectional Studies

Reviewer_____________________________________Date___________________________

Author _____________________________________Year_________Record Number______

	Yes	No	Unclear	Not applicable
1. Were the criteria for inclusion in the sample clearly defined?	☑	☐	☐	☐
2. Were the study subjects and the setting described in detail?	☑	☐	☐	☐
3. Was the exposure measured in a valid and reliable way?	☑	☐	☐	☐
4. Were objective, standard criteria used for measurement of the condition?	☑	☐	☐	☐
5. Were confounding factors identified?	☑	☐	☐	☐
6. Were strategies to deal with confounding factors stated?	☑	☐	☐	☐
7. Were the outcomes measured in a valid and reliable way?	☑	☐	☐	☐
8. Was appropriate statistical analysis used?	☑	☐	☐	☐

Overall appraisal: Include ☐ Exclude ☐ Seek further info ☐

Comments (Including reason for exclusion)

Major criteria: ✓

Minor criteria: ✓

Inclusion criteria: 6/8 (At least 5 meet the major inclusion criteria ✓)
