# Supplementary material for: Prevalence of hyperthyroidism, hypothyroidism, and euthyroidism in thyroid eye disease: a systematic review of the literature
Source: Syst Rev. 2020 Sep 1;9:201. doi: 10.1186/s13643-020-01459-7 (PMC7465839; doi:10.1186/s13643-020-01459-7)
Supplement: Supplementary file 3 — Additional file 3. JBI quality tools results for final selected articles. [file 13643_2020_1459_MOESM3_ESM.docx]

**Annex 3. JBI quality tools results for final selected articles**

| **Author** | **Study design** | **Met criteria** | **Inclusion minimal %** |
| --- | --- | --- | --- |
| Ackuaku-Dogbe et al. 2017 | Cross-sectional | 62.50% | 62.50% |
| Expósito et al. 2012 | Cross-sectional | 87.50% | 62.50% |
| Bartley et al.1995 | Cross-sectional | 100% | 62.50% |
| Eckstein et al. 2009 | Cohort | 70% | 63.63% |
| Jang et al. 2012 | Case control | 80% | 80% |
| Kashkouli et al. 2011 | Cross-sectional | 62.50% | 62.50% |
| Khoo et al. 2000 | Cohort | 72.72% | 63.63% |
| McKeag et al. 2007 | Cross-sectional | 87.50% | 62.50% |
| Medghalchi et al. 2018 | Cross-sectional | 87.50% | 62.50% |
| Mukasa et al. 2016 | Cross-sectional | 87.50% | 62.50% |
| Ponto et al. 2014 | Cross-sectional | 75% | 62.50% |
| Ponto et al. 2015 | Cross-sectional | 75% | 62.50% |
| Cozma et al. 2009 | Cross-sectional | 100% | 62.50% |
